# Bibliographical Notices

**Published:** 1839-06-01

**Authors:** 


### BIBLIOGRAPHICAL NOTICES.

*Bibliographical Memoir of Dr. James Johnson, Senior Editor of the London Medico-Chirurgical Review.* Medical Portrait Gallery. London: 1839.

Dr. James Johnson is at the head of the English medical periodical literature of the day. A lively and most agreeable writer, an acute critic, a laborious reviewer, a successful and experienced practitioner,—and, not a little distinguished for his general literary attainments, he unites qualifications which belong to no other professional *littérateur* whom we know. Dr. Johnson's popularity as a writer, is not confined to Great Britain. His review is reprinted and circulated widely in this country, admired as well for the vigour, originality, and sprightliness of the editor's style, as for the ability and erudition of his articles.

Dr. Johnson is, we learn from the memoir, a native of Ireland, where he was born in the year 1778. His professional career commenced in the navy, in 1798, where he roughed it for some years as a surgeon's mate. In 1801, he applied for a ship, armed with the following pithy certificate, from his preceptor, Mr. Wilson, of London:—"The *bearer* of this, Mr. James Johnson, has actually lived in the dissecting-room of Great Windmill street during the last six months. Examine him, and see whether he has studied in vain." He was immediately appointed to a sloop of war; afterwards, in 1803, to a frigate, in which he made a three years' cruise to India and China, to which we owe his work, "*The Influences of Tropical Climates on European Constitutions*, which he published in 1812. At the conclusion of the war in 1814, Dr. Johnson retired from the navy, and settled at Portsmouth; but, in 1818, ambition and indifferent health carried him to London. He started in the metropolis, almost without a friend, burthened with a wife and five children, and not worth five hundred pounds.

Dr. Johnson's works upon subjects of general literature, unquestionably assisted materially his advancement to practice and fame. In 1812, he published his work *On Tropical Climates*, which has since gone through five editions. In 1818, he started his Quarterly Review, which at once rose to a large and well-deserved circulation.

The *Essay on Indigestion*, published in 1823, has gone through nine editions, and, his biographer states, "brought his private practice to the highest point compatible with his health, which, of late years, has been remarkably good." In 1829, Dr. Johnson made a tour for health, through France, Switzerland, and Italy, which resulted in the publication of a most agreeable work, *Change of Air, or the Pursuit of Health.*

Dr. Johnson is now in the enjoyment of affluence, professional and literary reputation, and, still better, "in his domestic affairs, he has been most fortunate and happy." "In private practice, he is one of the most popular physicians of the metropolis. His manners are mild and kind to his patients, and he has the happy art of inspiring confidence in those whom he attends—an art which, like that of poetry, 'nascitur, non fit.'" We cordially rejoice in his well-merited success, and trust he may live long to enjoy it.

---

*Journal de la Société Medicale de la Nouvelle Orleans.* No. 1., prem. anneé. Janvier, 1839. *Journal of the Medical Society of New Orleans.* 1st Number. January, 1839.

This is the first number of a new periodical, published by the Medical Society of New Orleans. We have received no subsequent numbers, and do not know what has been the success of the work, as regards its subscription list.

It is published in the French language,—that is, the articles were written by the physicians composing what may be termed the French part of the profession at New Orleans; for there is a French portion of the medical profession, just as there is one of the citizens of New Orleans in general. This society, we perceive, is chiefly constituted by French physicians, who are all, or nearly all, graduates of the Parisian school. The prospectus, however, states that articles may be written either in the French or English languages as their authors may prefer, but they have not the liberty of requiring a translation. In Louisiana, as in Canada, the population, it is well known, is divided into two distinct portions, or races, partly from the difficulty of comprehending a foreign language, and partly from the national and political jealousy of the two divisions of citizens. Both French and English are used in the courts of law, and in the legislature of the state. Such an arrangement is attended with comparatively little inconvenience when it is adopted in verbal debates, but will, we are sure, be extremely awkward when applied to the purpose of a scientific journal. If the Journal of the Medical Society of New Orleans be destined for other portions of the United States than Louisiana, we are quite sure that the present form will be found to interfere with its circulation.

The present number contains an account of an operation for lithotomy; a case of delirium tremens; one of rupture of the uterus; another of two penetrating wounds of the chest; and several upon the use of chloride of soda as a substitute for quinine, in the treatment of intermittent. The number terminates with an interesting memoir upon the salts of morphia, by Dr. Bahier. He concludes that these salts should be given with great caution, and gradually increased, and that they cannot replace the older preparations of opium. "At an equivalent dose, the salts of morphia do not produce upon the economy an action identical with that of opium, and of its different preparations; they produce much more frequently the symptoms of narcotism, sometimes to an alarming degree."

The Journal does not contain extracts or summaries from European, or other sources, but is limited to the articles which we have just enumerated. An interesting series of articles will probably appear upon yellow fever, and other endemic diseases of New Orleans.

